# Expression Signatures of Long Noncoding RNAs in Adolescent Idiopathic Scoliosis

**DOI:** 10.1155/2015/276049

**Published:** 2015-09-01

**Authors:** Xiao-Yang Liu, Liang Wang, Bin Yu, Qian-yu Zhuang, Yi-Peng Wang

**Affiliations:** ^1^Department of Orthopedic Surgery, Peking Union Medical College Hospital, Chinese Academy of Medical Sciences & Peking Union Medical College, No. 1 Shuaifuyuan, Wangfujing, Dongcheng District, Beijing 100730, China; ^2^Department of Spine Surgery, Shandong Provincial Hospital Affiliated to Shandong University, Jinan, Shandong 250000, China

## Abstract

*Purpose*. Adolescent idiopathic scoliosis (AIS), the most common pediatric spinal deformity, is considered a complex genetic disease. Causing genes and pathogenesis of AIS are still unclear. This study was designed to identify differentially expressed long noncoding RNAs (lncRNAs) involving the pathogenesis of AIS. *Methods*. We first performed comprehensive screening of lncRNA and mRNA in AIS patients and healthy children using Agilent human lncRNA + mRNA Array V3.0 microarray. LncRNAs expression in different AIS patients was further evaluated using quantitative PCR. *Results*. A total of 139 lncRNAs and 546 mRNAs were differentially expressed between AIS patients and healthy control. GO and Pathway analysis showed that these mRNAs might be involved in bone mineralization, neuromuscular junction, skeletal system morphogenesis, nucleotide and nucleic acid metabolism, and regulation of signal pathway. Four lncRNAs (ENST00000440778.1, ENST00000602322.1, ENST00000414894.1, and TCONS_00028768) were differentially expressed between different patients when grouped according to age, height, classification, severity of scoliosis, and Risser grade. *Conclusions*. This study demonstrates the abnormal expression of lncRNAs and mRNAs in AIS, and the expression of some lncRNAs was related to clinical features. This study is helpful for further understanding of lncRNAs in pathogenesis, treatment, and prognosis of AIS.

## 1. Introduction

Adolescent idiopathic scoliosis (AIS), accounting for 90% of all idiopathic scoliosis (IS) cases, affects more than 2% of the pediatric population and results in more than 600,000 physician visits annually [1]. Approximately, 1.5% patients ultimately need surgical correction for their curvature [2]. However, the causes involved in AIS remain obscure.

Genetic twin studies and observation of familial aggregation have revealed significant genetic contribution to IS [3, 4]. A genetic survey study reported that an overall risk of IS in first-degree relatives was up to 11%, as compared to 2.4 and 1.4% in second- and third-degree relatives, respectively. These studies indicate that IS is an inheritable complex disease. Molecular studies found that some critical regions on chromosomes were potentially important for the occurrence of scoliosis [5, 6]. Genome-wide association studies of AIS in case-control cohorts also located candidate susceptibility genes, and these findings indicate that single nucleotide polymorphisms are valuable for the AIS prognosis [7]. However, inconsistent outcomes were observed among studies concerning the candidate genes in AIS [8–11]. Totally, the inheritance mode and pathogenic gene of AIS is uncertain until today.

Recent technological advances reveal that a major portion of the genome is being transcribed and that protein-coding sequences only account for a minority of cellular transcriptional output. MicroRNAs are endogenously expressed noncoding transcripts, shorter than 20 nucleotides. MicroRNA silences gene expression by targeting specific mRNAs on the basis of sequence recognition [12]. Long noncoding RNAs (lncRNAs) are transcripts longer than 100 nucleotides. lncRNAs in most cases mirror the features of protein-coding genes except that the former does not contain a functional open reading frame. A handful of studies have implicated that lncRNAs are involved in a variety of disease states [13] and altered lncRNA levels can result in aberrant expression of gene products that may contribute to many biological processes and human diseases [14–16]. Circulating levels of lncRNAs were reported to reflect local pathophysiological conditions [17, 18]. Expression level of RNA in peripheral blood has been used to evaluate local circumstance of spine [19, 20]. However, the expression of lncRNAs and their functions in AIS are still unknown.

In the present study, we measured lncRNAs expression in AIS patients using microarray and quantitative PCR (qPCR) analysis. Our results disclosed lncRNAs expression profiles and provide new information for further exploration of pathogenesis and prognosis of AIS.

## 2. Materials and Methods

### 2.1. Patients

Written informed consent was obtained from all participants. The study was approved by the Institutional Review Board of Chinese Academy of Medical Sciences and Peking Union Medical College Hospital. One hundred and twenty AIS patients (AIS) and twenty normal children (NC) were included in the study. Of these participants, four AIS patients and four children were used for microarray analysis. No significant difference was found between two groups in age, menarche, and Risser sign. Detailed information of the four pairs of participants is summarized in Table 1. The diagnosis of AIS was made only when other causes of scoliosis, including vertebral malformation, neuromuscular disorder, and syndromic disorders, were ruled out. Participants were screened with Adams' forward bend test. Cobb angle measured with a scoliometer was at least 10° [21]. Peripheral blood from each subject was added 3 times volume of Trizol LS reagent (Amboin) and immediately stored at −80°C until use.

### 2.2. RNA Extraction

Total RNA was extracted using Trizol LS reagent according to the instructions recommended by the manufacturer. The RNA purity and concentration were evaluated with NanoDrop ND-1000 spectrophotometer. RNA integrity was determined with 1% formaldehyde denaturing gel electrophoresis, which revealed a good quality (data not shown).

### 2.3. RNA Labeling and Hybridization

Double-stranded cDNAs (containing T7 RNA polymerase promoter sequence) were synthesized from 1 mg total RNA according to the manufacturer's instructions (Capitalbio) and labeled with a fluorescent dye (Cy3-dCTP). Labeled cDNA was denatured at 95°C for 3 min in hybridization solution. Agilent human lncRNA + mRNA Array V3.0 was hybridized in an Agilent Hybridization Oven overnight at 42°C and washed with two consecutive solutions (0.2% SDS, 2x SSC for 5 min at 42°C, and 0.2x SSC for 5 min at room temperature).

### 2.4. Microarray Analysis

The data from lncRNA + mRNA Array were used to analyze data summarization, normalization, and quality control using the GeneSpring software V11.5 (Agilent). The differentially expressed genes were selected if the change of threshold values was ≥2 or ≤−2 folds and if Benjamini-Hochberg corrected *P* values were <0.05. The data was normalized and hierarchically clustered with CLUSTER 3.0 software. The data were performed to be Tree Visualization with Java Treeview software (Stanford University School of Medicine, Stanford, CA, USA).

### 2.5. Construction of the Coding-Non-Coding Gene Coexpression (CNC) Network

The CNC network was constructed based on the correlation analysis between the differentially expressed lncRNAs and mRNAs. LncRNAs and mRNAs with Pearson correlation coefficients not less than 0.99 were selected to draw the network using open source bioinformatics software Cytoscape (Institute of Systems Biology in Seattle). In network analysis, red nodes represent the upregulated lncRNAs, dark blue nodes represent the downregulated lncRNAs, pink nodes represent the upregulated mRNAs and light blue nodes represent the downregulated mRNAs, circular nodes represent mRNAs, triangular nodes represent lncRNAs, dashed lines represent a positive correlation, and solid lines represent an inverse correlation.

### 2.6. qPCR

qPCR was performed using SYBR Premix Ex Taq on Thermal Cycler Dice TP800 instrument. Four lncRNAs (ENST00000440778.1, ENST00000602322.1, ENST00000414894.1, and TCONS_00028768), which revealed differentially expression, were evaluated in all participants. Total RNA (2 *μ*g) was reversely transcribed into cDNA using a PrimeScript RT reagent kit containing a gDNA Eraser (TaKaRa) according to the manufacturer's instructions. PCR was performed in 20 *μ*L of reaction system, including 10 *μ*L SYBR Premix Ex Taq (2x), 0.4 *μ*L of PCR forward primer (10 *μ*M), 0.4 *μ*L of PCR reverse primer (10 *μ*M), 1 *μ*L of cDNA, and 8.2 *μ*L of double-distilled water. Primers for lncRNAs and mRNA are listed in Table 2. All experiments were performed in triplicates. All samples were normalized to GAPDH. The median in each triplicate was used to calculate relative lncRNAs concentrations (ΔCt = Ct median lncRNAs-Ct median GAPDH). Folds change was calculated using 2^−ΔΔCt^ methods. The differences of lncRNAs expression between patients and control were analyzed using Student's *t*-test within SPSS (version 16.0 SPSS Inc.). A value of *P* < 0.05 was considered as statistically significant.

## 3. Results

### 3.1. LncRNAs Profiles

**Figure 1:**
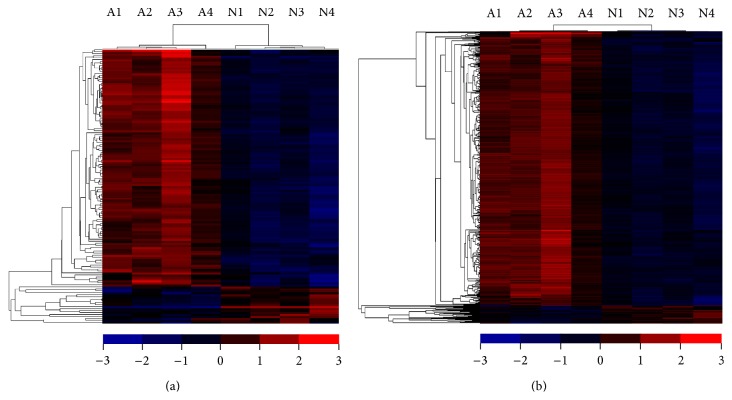
Heat maps of expression ratios (log2 scale) of lncRNAs (a) and mRNAs (b) between AIS patients and normal control. “Red” denotes high relative expression and “blue” denotes low relative expression.

Tens of thousands lncRNAs were examined in microarray. A total of 139 lncRNAs revealed significantly different expression in AIS group, compared to NC group (≥2-fold) (Table S1, see Supplementary Material available online at http://dx.doi.org/10.1155/2015/276049). Compared to NC group, a total of 118 lncRNAs were consistently upregulated or downregulated in all tested AIS samples. ENST00000440778.1 was the most significantly downregulated lncRNA (fold change = 9.780566), while NR_024075 (Log2 fold change = 3.8194413) was the most significantly upregulated one. The heat maps of the expression ratios of lncRNAs are shown in Figure 1(a).

### 3.2. mRNAs Profiles

The expression of coding transcripts (i.e., mRNAs) was examined with microarray containing 33,982 coding transcripts probes. Up to 546 mRNAs showed significant difference between AIS and NC groups (Table S2). A total of 512 mRNAs were upregulated in AIS, while 34 mRNAs were downregulated. The heat map of the expression ratios of mRNAs is shown in Figure 1(b). GO analysis showed that these mRNAs might be involved in bone mineralization, neuromuscular junction, skeletal system morphogenesis, and nucleotide and nucleic acid metabolism. Pathway analysis indicates that the dysregulated mRNAs are involved in cell adhesion molecules, Wnt signaling pathway, Toll-like receptor signaling pathway, MAPK signaling pathway, and so on. Coding RNAs related to major biological processes are listed in Table 3. These results support the viewpoint that AIS may be a genetic disease involving musculoskeletal system.

### 3.3. CNC Network

**Figure 2:**
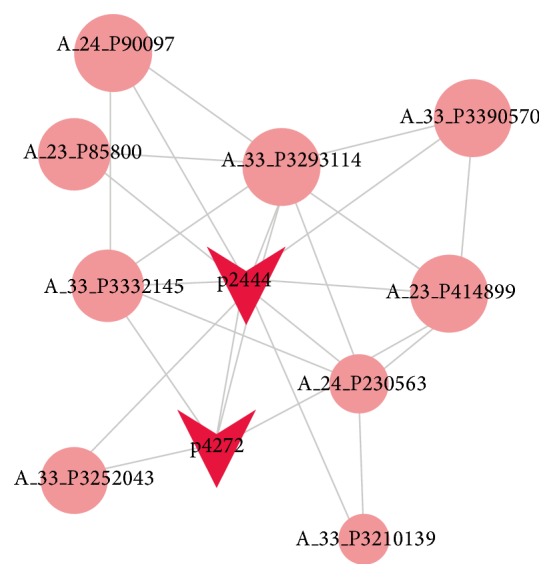
CNC network of lncRNA ENST00000602322.1 (labeled as p2444). Circular nodes represent mRNA; triangular nodes represent lncRNA. Detailed information of lncRNAs and mRNAs is listed in supplementary tables.

In the CNC network, there were 76 lncRNAs and 370 mRNAs. A total of 446 network nodes were gained and this was associated with 1121 pairs of coexpression of lncRNAs and mRNAs. The CNC network indicates that one mRNA is correlated with one to tens of lncRNAs and* vice versa*. The whole CNC network might implicate an interregulation between lncRNAs and coding RNAs in AIS. In particular, lncRNA ENST00000602322.1 was correlated with lncRNA ENST00000422231.2 and nine coding RNAs (Figure 2).

More and more evidences indicate that lncRNAs play important roles in gene expression. Thus, we explored the correlation between lncRNAs and mRNA. More than 1000 pairs of lncRNAs and mRNAs reached an absolute correlation coefficient greater than 0.99 and a False Discovery Rate less than 0.01. Target prediction indicated that seven lncRNAs (uc002ddj.1, uc021tnw, uc021zdc.1, HIT000067310, ENST00000577528.1, ENST00000602322.1, and TCONS_00001429) may influence the expression of related mRNAs. ENST00000602322.1 and HIT000067310 may regulate the mRNA expression of PCF11 and RCAN3, respectively. The two mRNAs are expressed in muscle system and encode proteins which regulate mRNA splicing and metabolism. These lncRNAs and mRNAs may play a role in the pathogenesis of AIS. Detailed information of lncRNAs and predicted mRNAs is listed in Table 4.

### 3.4. LncRNA Classification and Subgroup Analysis

Recently, some special classes or 14 clusters of lncRNAs have been identified with specific function in human cells. Numerous lncRNAs were found to be transcribed from the human homeobox transcription factors (HOX) clusters [22]. These lncRNAs are expressed in temporal and site-specific fashions and may be associated with distinct and diverse biological processes [23, 24], such as cell proliferation, RNA binding complexes, and muscle development. In the present study, our microarray probes targeted 407 discrete transcribed regions of four human HOX loci. None of them was differentially expressed between AIS and NC.

Harrow and his colleagues defined a set of lncRNAs with enhancer-like function in human cell lines [25]. Depletion of these lncRNAs led to decreased expression of their neighboring protein-coding genes. In the present study, 1896 lncRNAs with enhancer-like function were detected while 4 of them were differentially expressed in AIS. The differentially expressed enhancer-like lncRNAs and their nearby coding genes (distance, 300 kb) are shown in Table S3.

### 3.5. qPCR Validation

**Figure 3:**
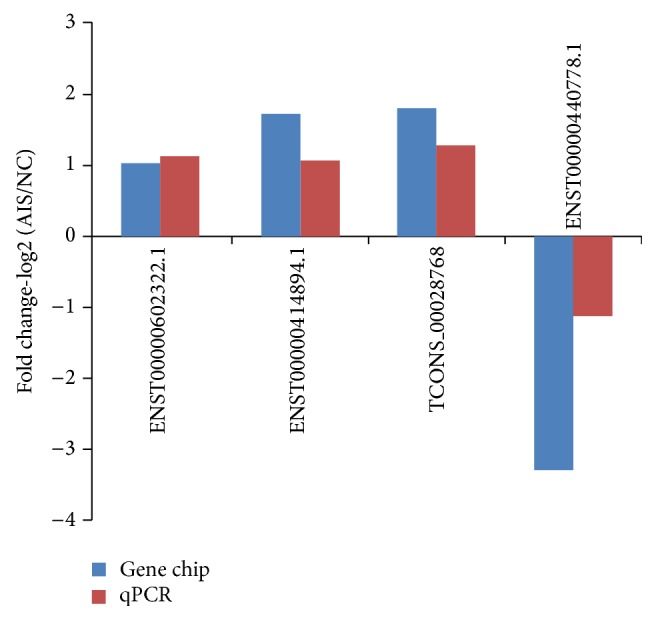
Comparison between microarray and qPCR results. The columns heights represent the log-transformed median fold changes (AIS/NC) in the AIS patients compared to normal control. The validation results of the four lncRNAs indicate that the microarray results match well the qPCR results.

Data from microarray were validated by qPCR in twenty pairs of samples. qPCR results revealed that ENST00000602322.1, ENST00000414894.1, and TCONS_00028768 were upregulated and ENST00000440778.1 was downregulated in AIS samples (*P* < 0.01), as compared to the control, which was consistent with those from microarray analysis (Figure 3).

### 3.6. Clinical Features of Validated lncRNAs

The correlation of lncRNA expression with clinical features was further analyzed. All 120 AIS patients were grouped according to age, height, menarche, classification, severity of scoliosis (measured by Cobb angle), and Risser grade (Table 5). No significant difference of lncRNAs expression was observed when patients were grouped according to menarche. The expression of ENST00000440778.1 and TCONS_00028768 was significantly different when grouped according to height (*P* < 0.05). The expression of ENST00000602322.1 was higher in younger patients (*P* < 0.01). Significant difference was also observed in ENST00000602322.1 expression between patients with single curve and double curves (*P* < 0.05). Lower expression of ENST00000414894.1 was observed in patients with Cobb angle greater than 40° (*P* < 0.05). The expression of ENST00000440778.1 was higher in patients with Risser grade ≤3 (*P* < 0.05).

## 4. Discussion

Although many clinical, epidemiological, and basic science researches were performed, the etiopathogenesis of AIS is still unclear [26]. Over the past decades, significant achievements have been made in profiling the molecular signatures in diseases using gene expression microarray [27]. Microarray has been recognized as a feasible and useful approach to explore pathogenesis and genetics of diseases and seek specific biomarkers [28]. Recently, hundreds of lncRNAs have been discovered, and altered lncRNAs expression has been considered to be correlated with cancer pathogenesis and muscles differentiation [29, 30]. However, the potential role of lncRNA expression in etiology and pathogenesis of AIS has not been systematically investigated.

To uncover the expression pattern of lncRNAs in AIS, we investigated the lncRNA expression signatures using microarray and found hundreds of differentially expressed lncRNAs in AIS patients. These lncRNAs may be involved in the development and progression of AIS. Based on the previous work and computer analysis, four lncRNAs (ENST00000440778.1, ENST00000602322.1, ENST00000414894.1, and TCONS_00028768) were selected to validate the consistency using qPCR. The expression of ENST00000440778.1 was downregulated in microarray and qPCR. But the expression gap between AIS and normal control was narrower in qPCR than in microarray. The difference may result from selection bias. Samples used in microarray analysis may lack representativeness, and ENST00000440778.1 expression in microarray analysis was not completely coincident between participants. Besides, it is necessary to investigate the specific lncRNAs in bigger size of samples.

In the present study, the differentially expressed mRNAs in AIS patients involve musculoskeletal development processes, including bone mineralization, neuromuscular junction, skeletal system morphogenesis, and nucleotide and nucleic acid metabolism. Pathway analysis indicates that the dysregulated mRNAs are related to cell adhesion molecules, Wnt signaling pathway, Toll-like receptor signaling pathway, MAPK signaling pathway, and so on. Precious studies have revealed the relationship between synapse formation, bone mineralization, and Wnt signaling pathway [31–33]. MAPK signaling pathway was also reported to be related to osteoblast differentiation and intervertebral disc cells degeneration [34, 35]. These biological processes and signaling pathway may play a significant role in the musculoskeletal system and pathogenesis of AIS.

More and more evidences reveal that lncRNAs act in both* cis* and* trans* [36, 37]. LncRNA ENST00000602322.1 locates on chromosome 11q and is adjacent to Pcf11 (Protein 1/Cleavage Factor 1). Pcf11 participates in transcription by coupling pre-mRNA [38]. Pcf11 is also found to play a role in transcription initiation, elongation, and mRNA export from nucleus to cytoplasm [39–41]. Given the key role of Pcf11 in transcription, it may not be surprising that ENST00000602322.1 may relate to the pathogenesis of AIS. The more detailed mechanisms of ENST00000602322.1 in transcription and translation need further investigation.

ENST00000440778.1 was upregulated 9.78-fold in AIS. Its expression was even higher in each AIS patient than that in healthy participants. Little is known about the function of ENST00000440778.1. However, its potential role in AIS pathogenesis was implied not only by its expression change but also by the clinical data from different height and Risser sign.

Our findings demonstrate differential lncRNA expression patterns in AIS when grouped according to different clinical features. This suggests potential significance in treatment and prognosis evaluation. Clinically, classification and curvature angle are important in evaluating surgical treatment and making operation plan in AIS [42, 43]. Onset time and Risser grade also play important roles in evaluating the progression of scoliosis [44]. It has been reported that lncRNAs are more specific than protein-coding mRNAs [45] and are easier to be detected in the blood samples of neoplastic and nonneoplastic patients using conventional PCR method [17, 18]. The differential expression of lncRNAs is potentially valuable in development of specific PCR markers and in providing more support on treatment and prognosis.

Several limitations exist in this study. First, although the different expression patterns of identified lncRNA genes suggest potential function in AIS pathogenesis, direct supporting evidence is lacking. Second, only four pairs of samples were used in microarray analysis. This may lose some important information and decrease the accuracy of biomarker selection. Third, RNA expression in peripheral blood was tested in our study. Regarding the idiopathic scoliosis being a musculoskeletal disease, tissue sample from musculoskeletal system may be more ideal targets. To accurately and comprehensively elucidate the role of lncRNAs in AIS, more comprehensive studies and laboratory and clinical researches are needed.

In summary, to the best of our knowledge, this is the first study that describes the expression profiles of human lncRNAs in AIS using microarray. Altered lncRNAs may play a potential role in the pathogenesis and/or development of this musculoskeletal disease. More work will be needed to confirm whether these lncRNAs play an essential role in the pathogenesis, treatment, and prognosis of AIS.

## Supplementary Material

Tens of thousands lncRNAs and mRNAs were examined in microarray, hundreds of which were differently expressed in AIS group compared to NC group. Detailed information of differently expressed lncRNAs and mRNAs was listed Table S1 and Table S2, respectively. Table S3 contains the differentially expressed enhancer-like lncRNAs and their nearby coding genes (distance < 300 kb).

## Figures and Tables

**Table 1:** Detailed information of the four pairs of participants.

Number	Group	Sex	Height	Age (years)	Illness history (years)	Menarche	Classification	Cobb angle (°)	Risser sign
A1	AIS	F	155	13.7	1	N	PUMCIIc2	50	0
A2	AIS	F	167	15.9	2	Y	PUMCIIc2	40	4
A3	AIS	F	158	15.0	1	Y	PUMCIb	44	5
A4	AIS	F	159	14.2	3	Y	PUMCIIc1	44	3
N1	NC	F	163	17.9	0	Y	—	0	5
N2	NC	F	163	13.2	0	Y	—	0	3
N3	NC	F	158	12.3	0	N	—	0	0
N4	NC	F	170	15.5	0	Y	—	0	4

**Table 2:** Primers used for qPCR.

lncRNAs	Sense primer (5′-3′)	Antisense primer (5′-3′)	Product (bp)
ENST00000602322.1	accttcccacctaccagtct	ctggggaaaccaccatagctt	150
ENST00000440778.1	acttggttgcttttccccaca	gctttgggcttggaaagatgg	107
ENST00000414894.1	agccgccctattcagttcac	tccaagttccgagttgtggg	148
TCONS_00028768	gcaaacctggaaatctcggc	ggccgcagacatcatcttct	142
GAPDH	ctataaattgagcccgcagcc	gcgcccaatacgaccaaatc	154

**Table 3:** Genes biological processes.

Biological processes	Gene symbol	Gene name	Entrez gene ID	Fold changes
Nervous system development	LTA	“Phosphoinositide-3-kinase, catalytic, delta polypeptide”	5293	2.1513674
	TP53	Tumor protein p53	7157	2.0308032
	MSH2	“mutS homolog 2, colon cancer, nonpolyposis type 1 (E. coli)”	4436	2.0266845
	BCL2	BCL2-associated athanogene 3	9531	2.130905
	TP53	Tumor protein p53	7157	2.0308032
	NCL	Nuclear RNA export factor 1	10482	2.00108
	PAFAH1B1	“Platelet-activating factor acetylhydrolase 1b, regulatory subunit 1 (45 kDa)”	5048	2.1079702
	OLIG1	Oligodendrocyte transcription factor 1	116448	2.129439
	PKD1	Polycystic kidney disease 1 (autosomal dominant)		2.18849
	BTG2	“BTG family, member 2”	7832	2.7636645

Skeletal morphogenesis and development	BCL2	B-Cell CLL/lymphoma 2	596	2.252202
	IL6ST	“Interleukin 6 signal transducer (gp130, oncostatin M receptor)”	3572	2.2222419
	MYC	v-myc myelocytomatosis viral oncogene homolog (avian)	4609	2.768515
	TPP1	Tripeptidyl peptidase I	1200	2.0187664
	AES	Aminoterminal enhancer of split	166	2.2728803
	ALOX15	Arachidonate 15-lipoxygenase	246	2.1062436

Muscle development and function	BCL2	B-Cell CLL/lymphoma 2	596	2.252202
	FLI1	Friend leukemia virus integration 1	2313	2.2254617
	UTRN	Utrophin	7402	2.1682687
	UTS2	Urotensin 2	10911	2.469184
	HIF1A	“Hypoxia inducible factor 1, alpha subunit (basic helix-loop-helix transcription factor)”	3091	2.028285
	TGFBR2	“Transforming growth factor, beta receptor II (70/80 kDa)”	7048	2.1115046
	TTN	Titin	7273	2.7300012
	LEF1	Lymphoid enhancer-binding factor 1	51176	3.1550286
	HIF1A	“Hypoxia inducible factor 1, alpha subunit (basic helix-loop-helix transcription factor)”	3091	2.028285
	FAM65B	“Family with sequence similarity 65, member B”	9750	2.247876
	UTRN	Utrophin	7402	2.1682687

Notch signaling pathway	ADAM17	ADAM metallopeptidase domain 17	6868	2.0420005
	NOTCH2	Notch 2	4853	2.1405845
	CTBP1	C-Terminal binding protein 1	1487	2.050679

MAPK signaling pathway	TAB2	TGF-beta activated kinase 1/MAP3K7 binding protein 2	23118	2.1040483
	SOS1	Son of sevenless homolog 1 (Drosophila)	6654	2.0901704
	DUSP16	Dual specificity phosphatase 16	80824	2.1876025
	MAP3K1	Mitogen-activated protein kinase kinase kinase 1	4214	2.343032
	RPS6KA3	“Ribosomal protein S6 kinase, 90 kDa, polypeptide 3”	6197	2.0745454
	CD14	CD14 molecule	929	2.3363564
	DUSP6	Dual specificity phosphatase 6	1848	2.2230558
	ARRB1	“Arrestin, beta 1”	408	2.004528
	TGFBR2	“Transforming growth factor, beta receptor II (70/80 kDa)”	7048	2.1115046
	AKT3	“v-akt murine thymoma viral oncogene homolog 3 (protein kinase B, gamma)”	10000	2.0013347
	MYC	v-myc myelocytomatosis viral oncogene homolog (avian)	4609	2.768515
	TP53	Tumor protein p53	7157	2.0308032
	NFKB1	Nuclear factor of kappa light polypeptide gene enhancer in B-cells 1	4790	2.0809784

Wnt signaling pathway	CAMK2G	Calcium/calmodulin-dependent protein kinase II gamma	818	2.1046312
	TCF7	“Transcription factor 7 (T-cell specific, HMG-box)”	6932	2.6018746
	PPARD	Peroxisome proliferator-activated receptor delta	5467	2.1376982
	TCF3	“Transcription factor 7 (T-cell specific, HMG-box)”	6932	2.8202255
	CAMK2D	Calcium/calmodulin-dependent protein kinase II delta	817	2.5258021
	PLCB2	“Phospholipase C, beta 2”	5330	2.2920265
	NFATC1	“Nuclear factor of activated T-cells, cytoplasmic, calcineurin-dependent 1”	4772	2.0340881
	MYC	v-myc myelocytomatosis viral oncogene homolog (avian)	4609	2.768515
	TP53	Tumor protein p53	7157	2.0308032
	LEF1	Lymphoid enhancer-binding factor 1	51176	3.1550286
	CTBP1	C-Terminal binding protein 1	1487	2.050679
	PIAS4	“Protein inhibitor of activated STAT, 4”	51588	2.0654333
	AES	Aminoterminal enhancer of split	166	2.2728803
	INVS	Inversin	27130	2.2433844
	LRRFIP2	Leucine rich repeat (in FLII) interacting protein 2	9209	2.1049194

Gene Ontology and Pathway analysis was performed for differentially expressed mRNAs. mRNAs involving musculoskeletal development are listed in their gene groups with associated Entrez Gene ID and fold changes per gene.

**Table 4:** Correlation between lncRNAs and mRNAs.

Source^a^	lncRNA	mRNA	Gene symbol	Correlation	*P* value	*cis*-Regulation^b^
p33784	uc002ddj.1	A_21_P0011418	PKD1	0.9967847	8.29*E* − 08	Sense
p33788	uc021tnw.1	A_24_P282108	ZZEF1	0.9976724	3.15*E* − 08	Sense
p26337	uc021zdc.1	A_23_P122615	PNISR	0.9907335	1.98*E* − 06	Sense
p26595	HIT000067310	A_23_P391275	RCAN3	0.9967367	8.67*E* − 08	Intergenic
p112	ENST00000577528.1	A_23_P391275	RCAN3	0.9965706	1.01*E* − 07	Intergenic
p2444	ENST00000602322.1	A_33_P3210139	PCF11	0.9932224	7.74*E* − 07	Antisense
p26595	HIT000067310	A_23_P35205	RCAN3	0.9900216	2.47*E* − 06	Sense
p29552	TCONS_00001429	A_23_P35205	RCAN3	0.9956033	2.12*E* − 07	Antisense

^a^Probe name of lncRNA. ^b^sense: the lncRNAs is a coding transcript exon on the same genomic strand; antisense: the lncRNA is transcribed from the antisense strand; intergenic: there are no overlapping or bidirectional coding transcripts nearby the lncRNA within 10 kbp.

**Table 5:** Expressions of lncRNAs in different clinical features.

Features		ENST00000602322.1	ENST00000440778.1	ENST00000414894.1	TCONS_00028768
Menarche age (y)	≤11	7.29 ± 0.82	4.74 ± 0.88	3.04 ± 0.52	5.51 ± 1.08
	>11	7.75 ± 1.20	5.09 ± 1.25	2.95 ± 0.75	5.62 ± 1.44

Height (cm)	≤160	7.49 ± 1.03	4.40 ± 0.71^∗^	2.98 ± 0.59	5.05 ± 0.99^∗^
	>160	7.73 ± 1.17	5.34 ± 1.45^∗^	2.99 ± 0.78	5.95 ± 1.63^∗^

Onset of AIS (y)	≤12	6.73 ± 0.60^∗∗^	4.92 ± 1.09	3.09 ± 1.52	5.54 ± 1.19
	>12	7.86 ± 1.29^∗∗^	5.01 ± 1.17	2.87 ± 1.17	5.63 ± 1.43

Numbers of curves^a^	1	7.09 ± 0.74^∗^	4.95 ± 1.40	2.79 ± 0.66	5.81 ± 1.24
	2	7.98 ± 1.19^∗^	4.98 ± 0.95	3.10 ± 0.66	5.46 ± 1.34

Cobb angle (°)	≤40	7.60 ± 1.36	5.11 ± 1.70	2.47 ± 0.63^∗^	5.59 ± 1.60
	>40	7.58 ± 1.95	4.90 ± 0.72	3.38 ± 0.65^∗^	5.59 ± 1.15

Risser sign^b^	≤3	7.51 ± 1.12	4.33 ± 0.81^∗^	2.93 ± 0.60	5.37 ± 0.97
	>3	7.65 ± 1.07	5.21 ± 1.25^∗^	3.02 ± 0.72	5.76 ± 1.51

Relative lncRNAs levels were normalized to GAPDH (ΔCt = CtlncRNA − CtGAPDH). Results are presented as mean ± standard deviation. ^∗^ *P* < 0.05, ^∗∗^ *P* < 0.01. ^a^Curves were counted and classified into single, double, and triple curves according to the apex number. ^b^Risser sign refers to the amount of calcification of the human pelvis as a measure of maturity. On a scale of 5, it gives a measurement of ossification progression; the grade of 5 means that skeletal maturity is reached.
